# Genetic and Epigenetic Regulation Networks: Governing from Cardiovascular Development to Remodeling

**DOI:** 10.1155/2017/4135956

**Published:** 2017-06-01

**Authors:** Junjie Xiao, Dragos Cretoiu, Zhiyong Lei, Saumya Das, Xinli Li

**Affiliations:** ^1^Cardiac Regeneration and Ageing Lab, Experimental Center of Life Sciences, School of Life Science, Shanghai University, Shanghai 200444, China; ^2^Victor Babes National Institute of Pathology, 050096 Bucharest, Romania; ^3^Division of Cellular and Molecular Biology and Histology, Carol Davila University of Medicine and Pharmacy, 050474 Bucharest, Romania; ^4^Laboratory of Experimental Cardiology, University Medical Centre Utrecht, 3508 GA Utrecht, Netherlands; ^5^Cardiovascular Division of the Massachusetts General Hospital and Harvard Medical School, Boston, MA 02215, USA; ^6^Department of Cardiology, The First Affiliated Hospital of Nanjing Medical University, Nanjing 210029, China

The development of the cardiovascular system and its remodeling in response to stress are complex processes under the intricate regulation of multiple molecules. Even subtle perturbations of these regulation networks have been implicated in cardiovascular diseases (CVD). Although significant efforts over the past few decades have helped reduce the mortality, CVD remains a major contributor to overall mortality and morbidity globally. Refining our understanding of the underlying molecular regulation networks may continue to contribute to the prevention and treatment of CVD.

Recent research has highlighted a number of genetic and epigenetic factors implicated in postnatal cardiac development and remodeling. This editorial entitled “Genetic and epigenetic regulation networks governing cardiovascular development and remodeling” encapsulates these recent findings. The selected papers in this issue will improve our understanding of the molecular mechanisms in cardiovascular development and remodeling and shed light on clinical diagnosis and treatment of CVD.

In this issue, genetic regulation of CVD is described by two groups. O. Irtyuga et al. identified six NOTCH1 variants in patients with aortic stenosis. Interestingly, they also showed that NOTCH1 mutations in aortic stenosis were associated with dysregulation of regulators of osteoprotegerin, receptor activator of nuclear factor *κ*B, and its ligand. S. Simpson et al. identified three SNPs associated with dilated cardiomyopathy in Irish Wolfhounds and confirmed that combining three loci analysis better predicts the incidence of diseases than individual genetic factors alone.

As central epigenetic factors, microRNAs (miRNAs, miRs) play vital roles in posttranscriptional regulation of gene expression and multiple miRNAs have been found essential for normal development and pathological diseases. Since a single miRNA could target several genes, an individual miRNA could affect many CVD. In this research topic, R. Y. Cao et al. provide a comprehensive overview of emerging roles of miR-155 in coronary artery disease, aneurysm formation, heart failure, and diabetic heart disease. S. Ding et al. reviewed the roles of miR-222 both in physiological process and pathological CVD. H. Gu et al. summarized the current understandings about miR-17-92 cluster in common cardiac diseases, as well as cardiac differentiation and proliferation during cardiac embryonic development. All three groups point out that targeting miRNAs such as miR-155, miR-222 and miR-17-92 cluster might be promising therapeutic targets of CVD, although the side effects and safe application in clinical treatment still need to be further addressed.

Accumulating evidence suggests that specific disease processes have a distinct miRNA expression profile. Through microarray analysis of patients and normal controls, H. Wang et al. identified 92 differently expressed miRNAs in patients with calcific aortic valve disease and J. Shi et al. identified 20 miRNAs differentially expressed in degenerative aortic stenosis patients. These two research articles provide evidence that distinct miRNA expression pattern indeed exists in individuals with different CVD. Therefore, miRNAs could serve as potential biomarkers for identifying patients with certain CVD. J. Wang et al. showed that circulating miR-146a increased in patients with good coronary collateral circulation in coronary artery disease and could be used as a potential biomarker for discriminating patients with poor or good coronary collateral circulation. Furthermore, as the constituents of exosomes, miRNAs play important roles in intercellular communication. L. Shao et al. showed that mesenchymal stem cell (MSC) and MSC derived exosomes (MSC-Exo) have similar miRNA expression profile and MSC-Exo are superior to MSCs in preserving myocardial function, indicating MSC-Exo as a novel therapeutic strategy for myocardial infarction. In contrast to pathological hypertrophy, exercise training induced physiological hypertrophy is favorable for cardiac function. J. Xu et al. predicted miRNAs and miRNA related biological pathways contributing to exercise-induced physiological cardiac hypertrophy using bioinformatics methods. In addition to cardiovascular remodeling, overwhelming studies have showed the important roles of miRNAs in postnatal heat development. P. Yu et al. showed the expression of seven cardiac or muscle specific miRNAs were changed in response to the time after birth in mice heart tissue, improving our understanding of cardiac development and providing new potential targets for treatment of heart diseases.

Circular RNAs (circRNAs), a novel type of epigenetic regulators, are becoming a research hotspot and play essential roles in the regulation of gene expression. X. Fan et al. summarized the current knowledge of the biogenesis, properties, and functions of circRNAs, especially their roles in CVD. This review lays the foundation for further study of circRNA function and indicates the great potential values of circRNA as diagnostic or prognostic biomarkers in various cardiac diseases.

Collectively, this research topic provides a thorough and updated summary about genetic and epigenetic regulation in cardiovascular development and remodeling and sets the stage for the related in-depth studies and clinical investigations.

*Junjie Xiao* *Junjie Xiao* *Dragos Cretoiu* *Dragos Cretoiu* *Zhiyong Lei* *Zhiyong Lei* *Saumya Das* *Saumya Das* *Xinli Li* *Xinli Li*

